# Characterization and Therapeutic Potential of Bacteriophage-Encoded Polysaccharide Depolymerases with β Galactosidase Activity against *Klebsiella pneumoniae* K57 Capsular Type

**DOI:** 10.3390/antibiotics9110732

**Published:** 2020-10-25

**Authors:** Nikolay V. Volozhantsev, Anna M. Shpirt, Alexander I. Borzilov, Ekaterina V. Komisarova, Valentina M. Krasilnikova, Alexander S. Shashkov, Vladimir V. Verevkin, Yuriy A. Knirel

**Affiliations:** 1 State Research Center for Applied Microbiology and Biotechnology, 142279 Obolensk, Moscow Region, Russia; borzilov@obolensk.org (A.I.B.); ekaterina20009@mail.ru (E.V.K.); krasv55@mail.ru (V.M.K.); lab-mdgip@mail.ru (V.V.V.); 2 N.D. Zelinsky Institute of Organic Chemistry, Russian Academy of Sciences, Leninsky Prospekt 47, 119991 Moscow, Russia; asya@ioc.ac.ru (A.M.S.); shash@ioc.ac.ru (A.S.S.); knirel@ioc.ac.ru (Y.A.K.)

**Keywords:** bacteriophage, polysaccharide depolymerase, *Klebsiella pneumoniae*, antimicrobial therapy

## Abstract

Bacteriophages and phage enzymes are considered as possible alternatives to antibiotics in the treatment of infections caused by antibiotic-resistant bacteria. Due to the ability to cleave the capsular polysaccharides (CPS), one of the main virulence factors of *Klebsiella pneumoniae*, phage depolymerases, has potential in the treatment of *K. pneumoniae* infections. Here, we characterized in vivo two novel phage-encoded polysaccharide depolymerases as therapeutics against clinical isolates of *K. pneumoniae*. The depolymerases Dep_kpv79 and Dep_kpv767 encoded by Klebsiella phages KpV79 (Myoviridae; Jedunavirus) and KpV767 (Autographiviridae, Studiervirinae, Przondovirus), respectively, were identified as specific β-galactosidases that cleave the *K. pneumoniae* K57 type CPS by the hydrolytic mechanism. They were found to be highly effective at combating sepsis and hip infection caused by *K. pneumoniae* in lethal mouse models. Here, 80–100% of animals were protected against death by a single dose (e.g., 50 μg/mouse) of the enzyme injected 0.5 h after infection by *K. pneumoniae* strains of the K57 capsular type. The therapeutic effect of the depolymerases is because they strip the capsule and expose the underlying bacterium to the immune attack such as complement-mediated killing. These data provide one more confirmation that phage polysaccharide depolymerases represent a promising tool for antimicrobial therapy.

## 1. Introduction

Antibiotic resistance of pathogenic bacteria has become an increasingly pressing clinical issue worldwide. *Klebsiella pneumoniae* is a well-known Gram-negative bacterium notorious for contributing to multidrug resistance. So far, *K. pneumoniae* has been considered as an opportunistic pathogen, primarily causing different types of healthcare-associated infections in immunocompromised patients, including pneumonia, urinary and intestinal tract infections, and wound or surgical site infections [1,2]. However, in recent years, a new type of hypervirulent *K. pneumoniae* (hvKp) which causes community-acquired invasive life-threatening infections characterized by pyogenic liver abscesses complicated with meningitis and endophthalmitis, has emerged worldwide, especially in Southeast Asia [2,3,4].

*K. pneumoniae* cells produce a number of virulence factors including fimbrial adhesins, lipopolysaccharides (O antigen), siderophore iron acquisition systems, and a polysaccharide capsule (K antigen) [2]. An essential virulence factor and a defense barrier of *K. pneumoniae* are a polysaccharide capsule (CPS) that allows a bacterial cell to survive and spread inside the host overcoming the protective mechanisms of the immune system. The capsule impairs phagocytosis and opsonophagocytosis of the *K. pneumoniae* cells by immune cells hinders the bactericidal action of antimicrobial peptides and blocks complement components, such as C3, thus preventing complement-mediated killing [2,5,6]. There is a tremendous amount of structural variability among *K. pneumoniae* capsules, and at least 134 capsular types have been identified, including 77 K-types recognized by serological typing [7] and new K-types characterized by molecular genotyping and sequencing of the capsule synthesis locus (K-locus, KL) [8,9]. Of all CPS-types *K. pneumoniae*, K1, K2, K5, K20, K54, and K57 strains overproducing capsular polysaccharides are the most virulent (hvKp) human pathogens [10]. In addition, the CPS is recognized as a primary receptor for bacteriophages that possess capsule depolymerization activities [11]. These enzymatic activities allow phage to get access to the cell surface and bind to the secondary receptor. Bacteriophage-encoded polysaccharide-degrading enzymes, known as polysaccharide (PS) depolymerases, are highly specific enzymes, typically associated with the tail structure of the virions such as tail fibers, tailspikes, base plate or tail tubular proteins [12,13,14]. Since depolymerases target and destroy cell structures, which are important for bacteria survival and virulence, they have been suggested as potential anti-virulence agents to control and prevent bacterial infections. Recently, there have been several reports on the successful application of PS-depolymerases in the treatment of infections caused by *Escherichia coli* [15], *Pasteurella multocida* [16], *Pseudomonas aeruginosa* [17], *Acinetobacter baumannii* [18]. It was shown that depolymerases successfully rescued mice and *Galleria mellonella* larvae infected by *K. pneumoniae*, including hvKp strains [19,20,21,22].

As previously reported, a number of PS-depolymerases specific for *K. pneumoniae* capsular type K1 [19,23], K2 [24], K3, K21 [22], K5, K8, K30/K69 [21], K11, K25, K35, K64, KN5 [23], K63 [20], KN1, KN3, KN4, and K56 [25] have been isolated and characterized. Pan et al. [26] identified nine PS-depolymerases in a broad host range bacteriophage infecting *K. pneumoniae* and correlated the activities of the gene products to all host strains that belonged to capsule types K1, K11, K21, K25, K30/K69, K35, K64, KN4, and KN5. However, depolymerases, specific for *K.pneumoniae* of K57 capsule type, have not been characterized yet.

Previously, we reported on the isolation and genome characterization of bacteriophage KpV767 that can produce polysaccharide depolymerase and infect hypermucoviscous *K.pneumoniae* of K57 capsular type [24]. In this study, we describe a new lytic phage KpV79 against K57 capsule type *K. pneumoniae* and characterize two K57 specific polysaccharide depolymerases from phages KpV79 and KpV767.

## 2. Results and Discussion

### 2.1. Bacteriophages KpV79 and KpV767 Are Specific for Klebsiella pneumoniae of Capsular Type K57

**Figure 1:**
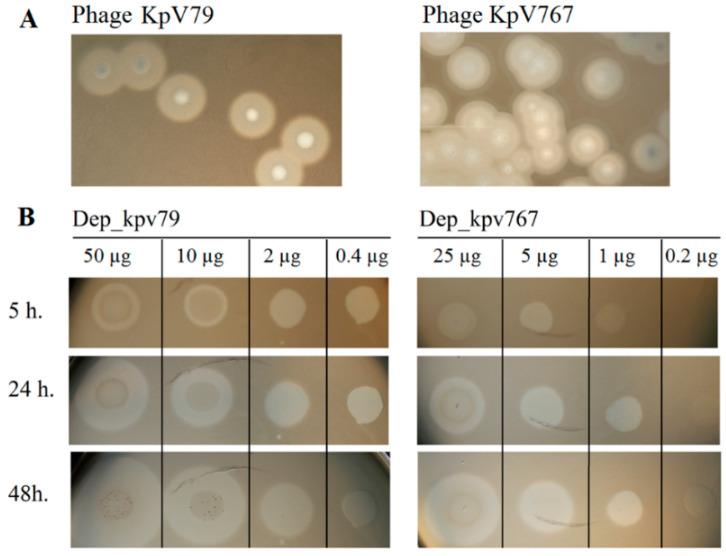
The activity of depolymerases Dep_kpv79 and Dep_kpv767 against *Klebsiella pneumoniae* strains. (**A**) Haloes surrounding phage KpV79 and KpV767 plaques, suggesting the presence of phage depolymerases. (**B**) Spot tests of purified depolymerases Dep_kpv79 and Dep_kpv767 on *K. pneumoniae* KPi8289 lawn. Aliquots (10 µL) of serial five-fold dilutions of Dep_79 and Dep_767 were spotted onto a plate containing the *K. pneumoniae* strain. The plates were observed for 5 h, 24 h, and 48 h of incubation at 37 °C.

Bacteriophages KpV767 and KpV79 were originally isolated from sewage in Russia in November 2015 and February 2016, respectively. We evaluated the lytic spectrum of KpV767 and KpV79 on a collection of *K. pneumoniae* clinical isolates (N = 250) including 21 strains of capsular type K57 (Table 1). Both phages multiplied and lysed *K. pneumoniae* capsular type K57 to form plaques with a halo expanding during incubation, which we suspected is related to a putative phage-derived depolymerase (Figure 1A). The phages did not multiply with plaque formation on some K57-type strains. However, in these cases, a translucent spot resembling the plaque halo was observed in the spot test by using undiluted phage suspension (Table 2). It should be noted that both phages were not active against others (non-K57 type) strains of the test collection (*n* = 229), as indicated by the absence of plaques of lysis or translucent spots on a bacterial lawn. These results indicate that phages KpV767 and KpV79 exhibit specificity to K57 capsular polysaccharides.

### 2.2. Genomes of Bacteriophages KpV79 and KpV767, Representing Distinct Taxonomic Groups, Contain Genes Encoding Polysaccharide Depolymerases with a High Level of Similarity at the Amino Acid Level

**Figure 2:**
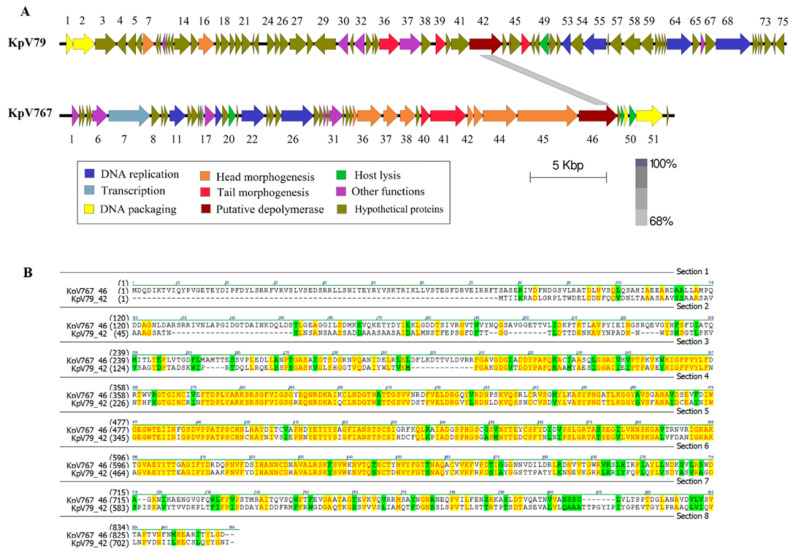
Genomic analysis of bacteriophages KpV79 and KpV767. (**A**) Open reading frame organization and comparison of phages genomes. (**B**) Comparison of amino acid sequences encoded by the PS-depolymerase genes kpvV79_42 and kpv767_46 from bacteriophages KpV79 and KpV767, respectively.

In a previous study [24], we presented the sequence of the bacteriophage KpV767 genome, consisting of 40,395 bp with a G + C content of 52.28%. The KpV79 phage genome has also been sequenced and analyzed. This phage has a linear double-stranded DNA genome of 47,760 bp. Its G + C content is 48.96%, and 75 open reading frames (ORF) are identified and annotated. In both phage genomes, putative functions were assigned to ORF products involved in phage DNA replication, transcription, packaging DNA into the capsid, host lysis, head, and tail morphogenesis (Figure 2).

In a number of bacteriophages, the proteins containing the depolymerase domain are usually located in tail spikes and tail fibers attached to the base plate, though they may also be located in other components of the virion, such as internal head or viral membrane [13]. Such proteins identified in genomes of the studied phages, as well as some hypothetical proteins with unknown functions, were analyzed for the presence of amino-acid and structural homology with carbohydrate-hydrolyzing enzymes in protein databases using PSI-BLAST and HHpred tools. To all appearances, the products of genes kpv79_42 and kpv767_46 of phages KpV79 and KpV767, respectively, are structural proteins with predicted PS depolymerase activity involved in the phage interaction with host bacterium. The proteins have significant homology within about 360 aa in the regions 309–671 aa of kpv767_46 protein and 177–539 aa of kpv79_42 protein, respectively (Figure 2).

A high level of similarity at the amino acid level in the central domain of proteins may indicate that these domains determine phage specificity and are responsible for the catalytic cleavage of polysaccharides. In contrast, differences in their N-terminal domains that are responsible for binding to phage particles reflect the fact that phages KpV79 and KpV767 are representatives of different families.

To confirm whether the predicted proteins have polysaccharide-degrading activity, we cloned genes kpv767_46 (phage KpV767) and kpv79_42 (phage KpV79) into the pET22b+ expression vector. His-tag fusion proteins Dep_kpv767 and Dep_kpv79 with a predicted molecular mass of 64 kDa and 77 kDa, respectively, were expressed and purified.

The activities of Dep_kpv767 and Dep_kpv79 were tested on 50 *K. pneumoniae* strains including 21 strains of K57 type. On spotting to a plate inoculated with *K. pneumoniae* K57 strains, the recombinant proteins generated a translucent spot resembling the plaque halo (Table 2; Figure 1B). At the same time, translucent spots were not formed on any bacterial lawn of non-K57 type *K. pneumoniae* strains. The presented results show that depolymerases Dep_kpv79 and Dep_kpv767, like parental phages KpV79 and KpV767, exhibit specificity to K57-capsulated *K. pneumoniae*.

### 2.3. Depolymerases Dep_767 and Dep_79 Inhibit the Adsorption of Corresponding Bacteriophages on a Capsule of Bacterial Cells

**Figure 3:**
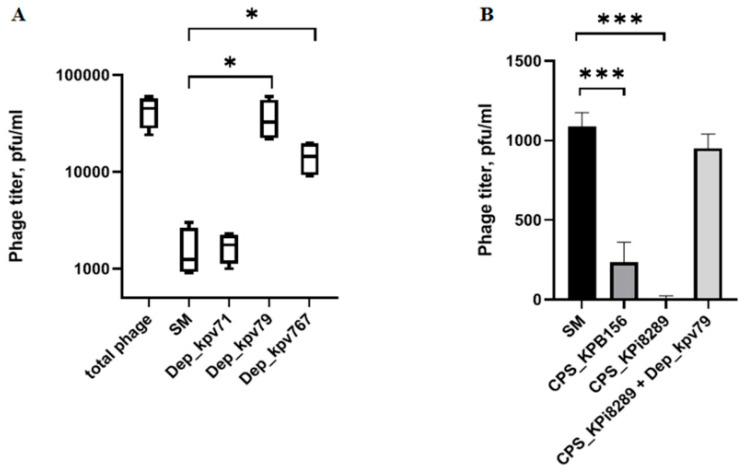
Phage KpV79 adsorption inhibition assay and phage inactivation by CPS. (**A**) From left to right, a titer of the total phage sample and titers of the free phage particles after the 5 min incubation with KpV79-sensitive *K. pneumoniae* KPi8289 cells in SM buffer and in the presence of Dep_kpv71, Dep_kpv79, and Dep_kpv767. (**B**) Purified CPS’s from *K. pneumoniae* KPB156 and KPi8289, as well as CPS KPi8289 pre-treated with PS-depolymerase Dep_kpv79, were added to identical phage samples and, after 10 min of incubation, aliquots were taken and titrated. Pure SM buffer was used as a negative control. Average titers from three independent experiments are shown and compared using Student’s *t*-test analysis. * *p* < 0.05, *** *p* < 0.001.

To study the interaction of bacterial viruses with surface receptors of a bacterial cell, quite simple methods are often used, such as inhibiting the adsorption of bacteriophages on the surface of bacteria by proteins of the phage receptor apparatus, including PS depolymerase, as well as phage inactivation by primary bacterial receptors such as capsule polysaccharides. We used a competition assay to assess the role of Dep_kpv79 and Dep_kpv767 in the KpV79 phage-cell interaction at the initial stages of the infection process. According to our preliminary results, at least 90% of the phage KpV79 are adsorbed to the *K. pneumoniae* cells in 5 min, and the phage latent period is about 30 min. The phage was added to the *K. pneumoniae* cells pre-treated with PS-depolymerases, and after 5 min of incubation at 37 °C, the titer of the phage that remained free in solution was measured. In one result, both K57 specific depolymerases Dep_kpv79 and Dep_kpv767 inhibited the phage-host binding. In the same conditions, K1-specific depolymerase Dep_kpv71 [24] did not affect phage adsorption (Figure 3A).

Primary bacterial receptors usually competitively inhibit phage adsorption on a bacterial cell. To evaluate the inhibitory effect of capsular polysaccharides on the bacteriophage KpV79, we conducted the phage inactivation by CPS. As a result, it turned out that the CPS of both strains can inactivate the bacteriophages. Furthermore, phage KpV79 inactivation did not occur if the CPS is pre-treated with Dep_kpv79 depolymerase (Figure 3B).

The data on specificity of the bacteriophages KpV79 and KpV767 to *K. pneumoniae* of the capsular type K57, as well as the inhibition of the phages by the K57-CPS, suggest that the capsule is the primary receptor for these phages. Both Dep_767 and Dep_79 inhibit the phage adsorption to *K. pneumoniae* cells, indicating that the phage and depolymerases compete for the same set of receptors on the host cell surface, that are capsular polysaccharides. These results are consistent with that Dep_kpv79 and Dep_kpv767 proteins were bioinformatics predicted as putative tail fibers/spikes that are responsible for host cell recognition. Based on adsorption experiments, we suppose that Dep_kpv79 and Dep_kpv767 proteins are responsible for the reversible binding and subsequent degradation of capsular polysaccharides of *K. pneumoniae* cells. This mechanism, followed by a stage of irreversible adsorption to the main receptors of the bacterial outer membrane, has been described for other capsule-specific phages [11].

### 2.4. Depolymerases Dep_kpv79 and Dep_kpv767 Specifically Cleave the β-Galactosidic Linkages in the K57 Capsular Polysaccharide of K. pneumoniae

Depending on the way of degradation of bacterial polysaccharides, phage-associated enzymes fall into the two main groups—glycoside hydrolases (EC 3.2.1.) and lyases (EC 4.2.2.)—which are divided into different subclasses [13]. Usually, the mechanism of the enzymatic action of phage enzymes is predicted according to amino-acid and structural homology with carbohydrate- degrading enzymes in protein databases. At the same time, only a few experimental data that relate to the enzymatic mechanisms of phage depolymerase action are available [17,27,28].

In this work, we elucidated the mechanism and characterized for the first time the product of cleavage of the *K. pneumoniae* K57 CPS by phage depolymerases Dep_kpv79 and Dep_kpv767.

**Figure 4:**
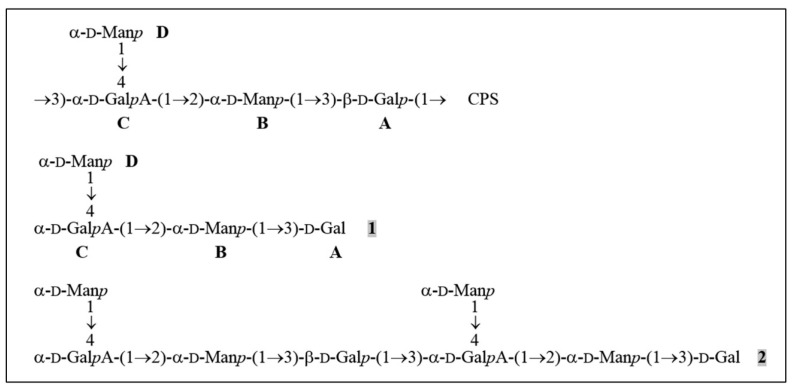
Structures of the K57 CPS of *K. pneumoniae* KPi8289 and oligosaccharides derived by cleavage of the CPS with depolymerase Dep_kpv79. CPS, tetrasaccharide repeat, containing two residues of D-mannose (units **B** and **D**) and one residue each of D-galactose (unit **A**) and D-galacturonic acid (unit **C**). 1 and 2, oligosaccharides obtained by cleavage of the CPS_KPi8289 with Dep_kpv79 (the same oligosaccharides were obtained by CPS splitting with Dep_kpv767).

The composition and structure of the K57 CPS of *K. pneumoniae* have been known [29]. CPS structure of the strain KPi8289 was confirmed by the assignment of the ^1^H and ^13^C NMR spectra of the CPS using two-dimensional ^1^H,^1^H COSY, ^1^H,^1^H TOCSY, and ^1^H,^13^C HSQC experiments (Table 3). The CPS is built up of branched tetrasaccharide repeats (K units) containing two residues of d-mannose (units B and D) and one residue each of d-galactose (unit A) and d-galacturonic acid (unit C) (Figure 4).

The CPS was cleaved with depolymerase Dep_kpv79 and the products were fractionated by gel permeation chromatography on Fractogel TSK HW-40S to give two oligosaccharides 1 and 2 (Figure 4). The same oligosaccharides were obtained by cleavage of the CPS_KPi8289 with Dep_kpv767 depolymerase (data not shown).

Analysis by negative ion mode high-resolution electrospray ionization mass spectrometry (HR ESI MS) showed that a lower oligosaccharide 1 corresponded to the K unit of the CPS and oligosaccharide 2 to a dimer of the K unit (the spectra showed [M-H]^−^ ion peaks for Hex_3_HexA_1_ and Hex_6_HexA_2_ at *m/z* 679.1841 and 1341.3834; calculated values *m/z* 679.1938 and 1341.3844, respectively).

The ^1^H and ^13^C NMR spectra of oligosaccharide 1 were assigned as described above for the CPS (Table 3). As in the K unit of the CPS, four monosaccharide residues (units A-D) were identified. Whereas the ^13^C NMR chemical shifts of B and D were essentially the same in the CPS and 1, those of units A and C were different. The monosaccharide residue at the reducing end of 1 that was identified as the Gal residue (unit A) occurred in two anomeric pyranose forms (δ_H_ 5.28 and 4.61, δ_C_ 93.6 and 97.3 for the α- and β-anomers; compare published data [30] δ_H_ 5.22 and 4.53, δ_C_ 93.18 and 97.37 for α-Gal*p* and β-Gal*p*, respectively). This residue is β-linked in the CPS. The GalA residue (unit C) is 3,4-disubstituted in the CPS and 4-substituted in 1, as followed from a marked displacement of the signal for C-3 of unit C from δ 78.4 in the CPS to δ 70.3 in 1.

Therefore, taking into account the CPS structure, oligosaccharide 1 has the structure shown in Figure 4. The same oligosaccharide has been obtained by mild acid hydrolysis of the K68 CPS of *K. pneumoniae,* which differs from the K57 CPS in the presence of a pyruvic acid acetal linked to the terminal Man residue [31].

The ^1^H and ^13^C NMR spectra of oligosaccharide 2 showed two series of signals, one corresponding to oligosaccharide 1 and the other to the K unit within the CPS. Particularly, there were signals for two units A, one β-linked as in the CPS and the other at the reducing end as in 1, and two units C, one 3,4-disubstituted and the other 4-substituted. Therefore, 2 represented a dimer of the K unit having the structure shown in Figure 4.

Thus, the data obtained indicated that depolymerases Dep_kpv79 and Dep_kpv767 are β-glycosidases that cleave specifically the β-d-Gal*p*-(1→3)-d-Gal*p*A (A→C) linkages in the CPS of *K. pneumoniae* KPi8289 by the hydrolytic mechanism.

### 2.5. Capsule Depolymerases Are Effective Therapeutics in Mouse Infection Models

Clinical manifestations of diseases caused by *K. pneumoniae* are extremely diverse [1,2]. Therefore, it is advisable to evaluate the therapeutic efficacy of bacteriophages on different laboratory models that differ in the route of infection and the infectious process development. We used two previously described mouse models of *K. pneumoniae* primary sepsis [32] and thigh soft tissue infection [33]. Two strains KPB156 and KPB550, differing in the degree of lysis by bacteriophages KpV79 and KpV767, were used to modeling *K. pneumoniae* infections in mice. As noted in Table 2, strain KPB550 was sensitive only to phage KpV79, whereas strain KPB156 was lysed by both phages with high efficiency of plating. Three groups of infected mice with 10 animals in each were used in each experiment: control (depolymerase untreated mouse) and two experimental (30 min after infection, the depolymerase Dep_kpv79 or Dep_kpv767 at a dose of 50 μg/mouse was injected intraperitoneally to mice of these groups).

**Figure 5:**
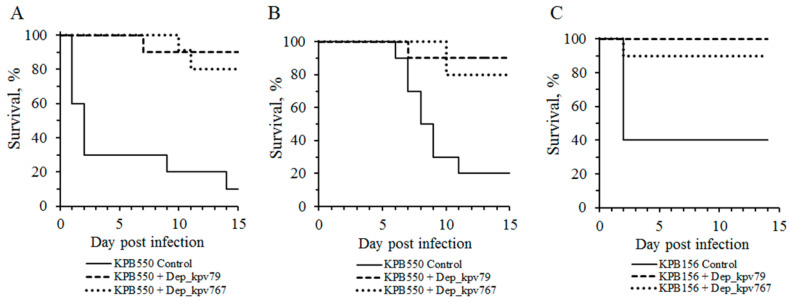
Protective effects of K57-specific depolymerases Dep_kpv79 and Dep_kpv767 of bacteriophages KpV79 and KpV767, respectively, in mice challenged with *K. pneumoniae* strains. Depolymerases (50 μg per mouse) were administered intraperitoneally 30 min after infection. (**A**) Primary sepsis caused by *K. pneumoniae* KPB550 (intraperitoneal injection). (**B**) Thigh soft tissue infection caused by *K. pneumoniae* KPB550 (intramuscular injection). (**C**) Primary sepsis caused by *K. pneumoniae* KPB156 (intraperitoneal injection).

As a result, nine of 10 (90%) and six of 10 (60%) mice infected with an intraperitoneal injection of *K. pneumoniae* KPB550 and KPB156 strains, respectively (primary sepsis model), and eight of 10 (80%) mice infected with an intramuscular injection of *K. pneumoniae* KPB550 (soft tissue infection model) died in the control groups (without PS-depolymerase treatment). In these cases, a culture of the infecting strain was isolated from all mice, those that died and those that survived. In contrast, 80% to 100% of the animals survived in the experimental groups treated with Dep_kpv79 and Dep_kpv767 depolymerase (Figure 5). It should be noted that no pathology was detected in the organs and tissues of the animals that survived the challenge with *K. pneumoniae*. In total, a culture of *K. pneumoniae* was isolated from five of the 53 surviving animals that were treated with Dep_kpv79 or Dep_kpv767 depolymerase.

The bacteria of strain KPB156 are less virulent compared to KPB550. Therefore, when mice were infected in the thigh muscles with a culture of strain KPB156 no death of animals was observed. However, seven of ten infected mice showed soft tissue edema at the injection site. After depolymerase treatment, edema was observed on day 3 in three of 10 mice, followed by its disappearance on day 7. In the control group, 30% of mice remained edematous for up to 10 days after infection.

Therefore, Dep_kpv79 and Dep_kpv767 depolymerases that cleave the K57 type CPS, have a therapeutic effect against *K. pneumoniae*–induced infections in mice.

Previously, a few works have been published on the in vivo therapeutic efficacy of CPS specific depolymerases against *K. pneumoniae*-induced infections [19,20,22]. Lin et al. demonstrated that treatment with the recombinant depolymerase provided increased survival in mice infected with *K.pneumoniae* of capsular type K1 [19]. Experiments by Majkowska-Skrobek et al. have shown that depolymerizing enzymes were effective against *K. pneumoniae* strains, representing K3, K21, and K63 capsular types, in a *G. mellonella* infecting model. In particular, the application of K3 and K21 specific depolymerases considerably increased the lifespan of *G. mellonella* larvae in a time- and *K. pneumoniae* strain–dependent manner [22]. The other depolymerase was effective against a native capsule of *K. pneumoniae* strains, representing the K63 type, and significantly inhibited Klebsiella-induced mortality of *G. mellonella* larvae in a time-dependent manner [20].

Our results on the therapeutic efficacy of two K57 specific depolymerases against *K. pneumoniae*-induced infection in mice provide one more confirmation that phage polysaccharide depolymerases represent a promising tool for antimicrobial therapy.

### 2.6. Depolymerases Increase Sensitivity to Serum-Complement-Mediated Killing

It is believed that the therapeutic efficacy of depolymerases is due to their antivirulence potential [20]. Since the polysaccharide capsule is one of the major virulence factors of *K. pneumoniae*, the CPS destruction makes bacteria less virulent and more susceptible to innate host defense including pathogen phagocytosis and complement-mediated killing [19,22,23,34].

**Figure 6:**
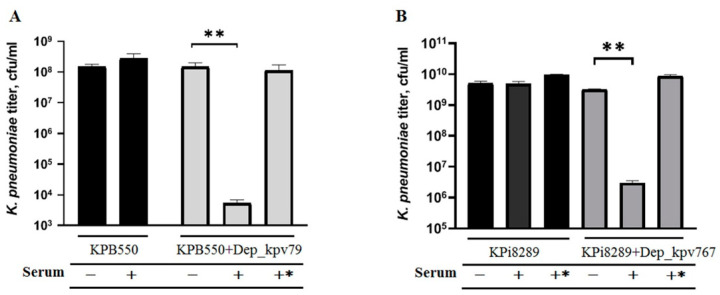
Serum sensitivity assay. Bacteria of serum-resistant *K. pneumoniae* strains KPB550 (**A**) and Kpi8289 (**B**) were pre-incubated with or without depolymerase Dep_kpv79 and Dep_kpv767, respectively (100 µg/mL, overnight at 4 °C). Mixtures were diluted 10 times, incubated for 3 h at 37 °C with 50% sheep serum (+), heat-inactivated serum (+*) or PBS (-), and then plated to determine colony-forming units. Average titers from three independent experiments are shown and compared by Student’s t-test analysis. ** *p* < 0.01.

In our work, the influence of the depolymerases on the *K. pneumoniae* serum resistance was studied. As shown in Figure 6, the depolymerases did not affect bacterial survival in the absence of serum. Also, serum alone did not affect the killing of *K. pneumoniae* strains KPB550 and KPi8289. However, treatment of *K. pneumoniae* cells with depolymerase Dep_kpv79 or Dep_kpv767 resulted in a decrease of the bacteria survival by four orders of magnitude after 3-h incubation. The pre-heated serum did not affect the viability of the *K. pneumoniae* cells. Therefore, increased bactericidal serum activity is associated with complement-mediated killing.

These results show that the depolymerases increase *K. pneumoniae* sensitivity to serum complement-mediated killing, consistent with other findings [19,22,34]. In addition, our data confirmed that the CPS is responsible for the complement resistance since other virulence factors of *K. pneumoniae* were unchanged after depolymerase treatment.

## 3. Materials and Methods

### 3.1. Bacterial Strains and Bacteriophages

Bacterial strains used in the experiments were obtained from the State Collection of Pathogenic Microorganisms and Cell Cultures (SCPM, Obolensk, Russia). All bacteria were routinely cultured in the Nutrient Medium No.1 (SRCAMB, Obolensk, Russia), or on LB broth agar (Difco Laboratories, Detroit, MI, USA) plates at 37 °C stationary or with shaking.

Bacteriophages were propagated using liquid cultures of *K. pneumoniae* sensitive strains as previously described [24]. Phage suspensions were titrated by a standard soft agar overlay method and stored at 4 °C until use.

### 3.2. Determination of the Host Range of Phage and Polysaccharide Depolymerase

Bacteriophages were screened against *K. pneumoniae* strains using the efficiency of the plating micro method [24]. PS depolymerase activity was assessed using the spot assay on *K. pneumoniae* strains of different capsular types and varying the enzyme concentrations. For *K. pneumoniae* lawn formation, 0.3 mL of bacterial suspension (~3 × 10^8^ CFU/mL) was mixed with 4 mL of soft agar (LB broth supplemented with 0.6% agarose) at 45 °C and poured onto the surface of the Nutrient Medium No.1 plate. The plate was incubated at 37 °C overnight. These bacterial lawns as well as lawns inactivated by chloroform vapor were used for depolymerase testing.

### 3.3. Analysis of the Phage Genomes

Phage genomes were sequenced using the Ion Torrent PGM (Life Technologies, Carlsbad, CA, USA), according to the manufacturer’s instructions. De novo assembly of sequence reads was performed using Newbler v.2.9. The correctness of assembly was checked using the SeqMan NGen software (DNAStar, Madison, WI, USA).

Protein-encoding genes and their putative functions were automatically predicted by RAST [35] and compared with known gene and protein sequences using the BLASTx and BLASTp algorithms [36] at the NCBI website. Linear genome comparisons of the phages and visualization of the coding regions were performed with Easyfig [37].

To identify putative polysaccharide depolymerase, a tail fiber, tail spike, and hypothetical proteins were analyzed by PSI-BLAST at the NCBI website and were screened through the HHpred web server [38].

The annotated genome sequences of bacteriophages KpV79 and KpV767 were deposited in the GenBank database and are available under accession numbers MF663761, and KX712070, respectively.

### 3.4. Cloning of CPS Depolymerase Genes and Preparation of Recombinant Proteins

Coding sequences for genes kpv79_42 (GenBank: ATI16495.1) and kpv767_46 (GenBank: AOZ65519.1) containing the putative depolymerase motifs were amplified from DNA of phages KpV79 and KpV767, respectively, by PCR using the following oligonucleotide primer pairs 79_XhoI: 5′-CTTACTCGAGGTTACCATAATACTGCAGAGAA-3′ and 79_NcoI: 5′-TATACCATGGACATTATCAAACGCGCAGAC-3′ (kpv79_42 gene cloning); 767_XhoI; 5′-TTAGTCCTCGAGATACGTGATGCGAGCCTC-3′ and 767_NcoI: 5′-CTTCACCATGGCAATGACCACAGAGTCTAG-3′ (kpv767_46 gene cloning).

The amplified genes were cloned into pET22b expression vector (Novagen, Madison, WI, USA) via the NcoI and XhoI sites by using *Escherichia coli* strain DH5α. The kpv79_42 gene was cloned entirely; the kpv767_46 gene was cloned without N-terminus fragment encoding the first 252 amino acid residues. Recombinant plasmids were verified by Sanger sequencing and then transformed into *E. coli* BL21 (DE3) for protein expression.

The production of recombinant proteins in LB broth (Difco Laboratories, Detroit, MI, USA) supplemented with 100 µg/mL ampicillin was induced by the addition of isopropyl-β-D-thiogalactopyranoside (IPTG) to the final concentration of 0.5 mM for 20 h at 22 °C. The recombinant proteins were separated from contaminating proteins by affinity chromatography using a Ni-iminodiacetic acid-Sepharose 5 mL column. The CPS-degrading activity of the recombinant proteins was assayed in the spot-test using *K. pneumoniae* strains of different capsular types [24].

### 3.5. Determination of the Structures of the Oligosaccharides Derived by the Treatment of K. pneumoniae CPS with Phage Depolymerases

Polysaccharides were isolated from *K. pneumoniae* KPi8289 cells by the modified water—phenol extraction method described previously [39].

Gel-permeation chromatography was carried out on a column (85 × 2.5 cm) of Fractogel TSK HW-40S (Merck, Germany). Elution was performed with 0.1% HOAc at a flow rate of 0.5 mL/min and monitored with a differential refractometer (Knauer, Germany).

^1^H and ^13^C NMR spectra were recorded on a Bruker Avance II 600 MHz spectrometer (Germany). Before measurement, samples were freeze-dried from 99.9% D_2_O and then examined in 99.95% D_2_O at 60 °C using sodium 3-(trimethylsilyl)propanoate-2,2,3,3-d_4_ (δ_H_ 0, δ_C_ −1.6) as the internal reference for calibration. Two-dimensional NMR spectra were obtained using standard Bruker software, and the TopSpin 2.1 program (Bruker, Germany) was employed to acquire and to process the data. A MLEV-17 spin-lock time of 60 ms was used in the TOCSY experiment.

HR ESI MS was performed in the negative ion mode using a micrOTOF II instrument (Bruker Daltonics). Oligosaccharide samples (~50 ng/μL) were dissolved in a 1:1 (*v/v*) water/acetonitrile mixture and injected with a syringe at a flow rate of 3 μL/min. The capillary entrance voltage was set at 3200 V, and the interface temperature at 180 °C. Nitrogen was used as the drying gas. The mass range was from *m/z* 50 to 3500 Da. Internal calibration was done with ESI Calibrant Solution (Agilent).

### 3.6. Phage Adsorption Inhibition Test and Phage Inactivation by CPS

The effect of PS-depolymerases Dep_767 and Dep_79 on the bacteriophage adsorption to *K. pneumoniae* cells was evaluated as described previously [40] with some modifications. In brief, a bacterial cell suspension (~2 × 10^8^ cfu/mL) in SM buffer (8 mM MgCl_2_, 100 mM NaCl, 50 mMTris-HCl pH 7.5) with added depolymerase (400 μg/mL) and a phage sample at 10^5^ pfu/mL were first brought to 37 °C. Then equal volumes of the phage and cell suspension were mixed. After 5 min incubation at 37 °C, bacterial cells with adsorbed phage were precipitated by centrifugation at 14,000 *g* for 1 min, and the titer of non-adsorbed phages was determined in the supernatant.

The phage inactivation by CPS was performed as follows. Phage samples (10^3^ pfu/mL) in SM buffer with added CPS at a final concentration of 1 μg/mL were incubated at 37 °C for 10 min. After incubation, the phage titer was assessed by the double agar layer technique.

### 3.7. In vivo K. pneumoniae Infection Models

Female white outbred mice (age 6–8 weeks) were used in experiments. Mice were housed under standard conditions of light, temperature, and water and food availability.

Two previously described mouse models of *K. pneumoniae* primary sepsis [32] and thigh soft tissue infection [33] were used to evaluate the therapeutic efficacy of PS-depolymerases. In the first case, *K. pneumoniae* strain KPB550 (LD_50_ = 6 × 10^6^ cfu) or KPB156 (LD_50_ = 1 × 10^8^ cfu) was used for intraperitoneal infection of mice (2 × 10^8^ cfu in a volume of 0.5 mL). In the second model, mice were injected intramuscularly (in the left thigh) with a culture of *K. pneumoniae* strains and at the same dose in a volume of 0.1 mL. In each case, control and experimental groups of mice were used (10 animals each). PS-depolymerases were injected into the mice of the experimental group half an hour after infection (once, intraperitoneal, 50 µg per mouse). Mice of the control groups were not treated with depolymerases. All animals were sacrificed two weeks after infection, and blood and parenchymal organs were examined for the presence of a *K. pneumoniae* culture. Mice that died during the experiment were also autopsied and examined for bacterial dissemination.

Mouse experiments were performed following the Directive 2010/63/EU of the European Parliament and of the Council of 22 September 2010 on the protection of animals used for scientific purposes. The experiments were approved by the Animal Ethics Committee of State Research Center for Applied Microbiology and Biotechnology.

### 3.8. Serum Complement Activity

A log-phase bacterial culture (~10^8^ cfu/mL) were preincubated with or without depolymerase (at the final concentration of 100 µg/mL) overnight at 4 °C. Mixtures were diluted 10 times, incubated for 3 h at 37 °C with 50% sheep serum, heat-inactivated serum (56 °C, 30 min) or phosphate-buffered saline (PBS), and then plated to determine colony-forming units. Each test was performed at least three independent experiments.

### 3.9. Statistical Analysis

Computation of data and statistical tests were performed using GraphPad Prism 8.0 software (GraphPad Software, Inc., La Jolla, CA, USA). Statistical significance was determined using a *t*-test. *p* < 0.05 was considered statistically significant.

## 4. Conclusions

In this work, we characterized two new PS-depolymerases Dep_kpv79 and Dep_kpv767 encoded by myovirus KpV79 and phage KpV767 of the Autographiviridae family both specific to *K. pneumoniae* K57. The depolymerases have a high level of similarity at the amino acid level in the central domain, which is responsible for the catalytic cleavage of polysaccharides. To the best of our knowledge, this is the first report of two identical depolymerases with the same CPS specificity derived from phages of a different family.

The phages recognize the K57 CPS as the primary receptor for adsorption, which is the first step in the phage infection process. These results were confirmed by a phage inactivation assay performed with the CPS isolated from *K. pneumoniae* strains of the K57 capsular type.

For the first time, we elucidated the mechanism and characterized the products of cleavage of the *K. pneumoniae* K57 CPS by depolymerases Dep_kpv79 and Dep_kpv767 encoded by phages with different tail structures. Both depolymerases were identified as specific glycosidases that cleave the CPS of *K. pneumoniae* strains by the hydrolytic mechanism.

Due to the ability to cleave the CPS, one of the main virulence factors of *K. pneumoniae*, depolymerases show a therapeutic effect in the treatment of *K. pneumoniae*–induced infections. Previously it has been reported that CPS-specific depolymerases successfully rescued mice and *G. mellonella* larvae infected by *K. pneumoniae* strains of capsular type K1 [19], K3, K21 [22], K63 [20], and K64 [34]. The Dep_kpv79 and Dep_kpv767 depolymerases were highly effective at combating sepsis and hip infection caused by *K. pneumoniae* in lethal mouse models. Both depolymerases exerted a therapeutic effect in mice infected with strains that differ in virulence and phage sensitivity. Indeed, 80–100% of animals were protected against death by a single dose (i.p., 50 μg/mouse) of the enzyme injected 0.5 h after infection by *K. pneumoniae* strains of K57 capsular type. The therapeutic effect of depolymerases is because they strip capsules and expose the underlying bacterium to immune attack such as complement-mediated killing.

Our study provides another confirmation that phage-borne depolymerases are able to target with high specificity such virulence factors as a bacterial capsule. Therefore, they represent a promising tool for typing and treatment of infections caused by bacterial pathogens.

## Figures and Tables

**Table 1:** General features of bacteriophages KpV79 and KpV767.

Characteristics	KpV79	KpV767
Isolation source	sewage	sewage
Isolation date	Feb-2016	Nov-2015
Genome length, bp	47,760	40,395
Total genes	75	52
Predicted PS depolymerase gene	kpv79_42	kpv767_46
The number of strains lysed by phage		
K57 type (*n* = 21)	18 ^b^ + 3 ^c^	10 ^b^ + 10 ^c^
Not-K57 type (*n* = 229) ^a^	0	0

^a^ Strains of capsular type K1, K2, K3, K13, K20, K22, K23, K24, K27, K28, K31, K39, K47, K54, K60, K62, K64, LK107 (*n* = 116), as well as 113 untyped strains of non-K57 type were tested. ^b^. The number of strains on which the bacteriophage multiply and form plaques with a translucent halo. ^c^. The number of strains on which the bacteriophage does not multiply but forms a translucent spot without plaque formation.

**Table 2:** The activity of bacteriophages KpV79 and KpV767 and their PS-depolymerases Dep79 and Dep767 against the *K. pneumoniae* strains of capsular type K57.

#	Strain	HV Phenotype	The Efficiency of Plating (EOP) ^a^		Spot-Test ^b^	
			KpV79	KpV767	Dep79	Dep767
1	KPB1106-2	−	H	H	++	+++
2	KPB156	+	H	H	++	+
3	KPB335	−	L	T	++	+
4	KPB500	−	L	-	+/−	−
5	KPB542-15	−	M	H	++	++
6	KPB550	−	L	T	++	++
7	KPB567	+	L	T	++	++
8	KPB584	−	T	T	++	+
9	KPB612-1	+	L	T	++	++
10	KPB690	+	L	T	+++	++
11	KPB697-1	−	T	T	++	++
12	KPB742	−	H	H	++	++
13	KPB757	+	L	T	+++	+++
14	KPB774-1	−	H	H	+++	+++
15	KPB811	+	T	T	++	++
16	KPBP1	−	M	H	+++	+++
17	KPi112	−	H	H	++	+
18	KPi4605	−	H	H	+++	+++
19	KPi4891	−	H	H	+++	+++
20	KPi8289	+	H	L	+++	+++
21	KPX4	−	H	T	++	+

^a^ Efficiency of plating (EOP) was calculated as the ratio of the PFU on the test strain to PFU on the host strain. “H”, high efficiency (EOP values 0.1–1); “M”, medium efficiency (EOP 0.001-0.01); “L”, low efficiency (EOP values 0.0001 or less); “T”, translucent spot without plaque formation; “–“, no lysis. ^b^ The result was recorded after 15 min (+++), an hour (++), and two hours (+) of incubation at 37 °C and subsequent incubation at 8 °C overnight (+/−). “−“, no activity.

**Table 3:** ^1^H and ^13^C NMR chemical shifts (δ, ppm) of the K57 CPS from *K. pneumoniae* KPi8289 and oligosaccharide 1 (OS) derived by cleavage of the CPS with depolymerase Dep_kpv79.

Residue	H-1	H-2	H-3	H-4	H-5	H-6
	*C-1*	*C-2*	*C-3*	*C-4*	*C-5*	*C-6 (6a, 6b)*
CPS						
→3)–β–δ–Γαλ*π*–(1→	4.66	3.64	3.72	4.01	3.63	3.77, 3.83
A	*105.7*	*71*	*78*	*65.9*	*76.3*	*62.5*
→2)–α–δ–Μαν*π*–(1→	5.21	4.05	4.02	3.77	4.01	3.75, 3,84
B	*96.2*	*81.2*	*71.6*	*68.4*	*74.1*	*62.3*
→3,4)–±–δ–Γαλ*π*A–(1→	5.21	4.07	4.16	4.63	4.57	
C	*102.1*	69.5	*78.4*	*79*	*72.2*	*174.7*
α-d-Man*p*-(1→	4.99	3.87	3.82	3.69	4.02	3.80, 3,89
D	*101.5*	*71.4*	*71.6*	*68*	*74*	*62.2*
OS						
→3)–α–δ–Γαλ*π*	5.28	3.91	3.94	4.19	n.f.	3.73–3.88
Aα	*93.6*	*68.1*	*74.5*	*66.3*	n.f.	*62.5*
→3)–β–δ–Γαλ*π*	4.61	3.56	3.73	4.12	3.65	3.73–3.88
Aβ	*97.3*	*71.7*	*77.9*	*65.8*	*76.3*	*62.4*
→2)–α–δ–Μαν*π*–(1→	5.22	4.02	4.03	3.78	3.84	3.73–3.88
B	*96.1*	*81*	*71.7*	*68.4*	*74*	*62.2*
→4)–α–δ–Γαλ*π*A–(1→	5.18	3.87	4.02	4.4	4.39	
C	*102.1*	*70.1*	*70.3*	*80.5*	*72.6*	n.f.
α-d-Man*p*-(1→	4.91	3.92	3.85	3.67	3.98	3.73–3.88
D	102.4	*71.5*	*71.5*	*68*	*74.2*	*62.2*

^13^ C NMR chemical shifts are italicized. n.f., not found.
